# Serological survey in wild boar (*Sus scrofa*) in Switzerland and other European countries: *Sarcoptes scabiei* may be more widely distributed than previously thought

**DOI:** 10.1186/s12917-018-1430-3

**Published:** 2018-03-27

**Authors:** Chloé Haas, Francesco C. Origgi, Sophie Rossi, Jorge R. López-Olvera, Luca Rossi, Raquel Castillo-Contreras, Anna Malmsten, Anne-Marie Dalin, Riccardo Orusa, Serena Robetto, Luciano Pignata, Santiago Lavín, Marie-Pierre Ryser-Degiorgis

**Affiliations:** 1 Centre for Fish and Wildlife Health, Vetsuisse Faculty, University of Bern, Länggass-Str. 122, Postfach, 3001 Bern, Switzerland; 2 Office National de la Chasse et de la Faune Sauvage, Unité Sanitaire de la Faune, Micropolis, la Bérardie, Belle Aureille, 05000 Gap, France; 3 Wildlife Ecology & Health group (WildEH) and Servei d’Ecopatologia de Fauna Salvatge (SEFaS), Departament de Medicina i Cirurgia Animals, Universitat Autònoma de Barcelona, 08193-Bellaterra, Barcelona, Spain; 4 Dipartimento di Scienze Veterinarie, Università di Torino, Largo Braccini 2, 10095 Grugliasco, Torino, Italy; 5 Sveriges lantbruksuniversitet, Institution för kliniska vetenskaper, Avdelning för reproduktion, Box 7054, 75007 Uppsala, Sweden; 6 National Reference Centre for Wildlife Diseases (CeRMAS), Istituto Zooprofilattico Sperimentale del Piemonte, Liguria e Valle d’Aosta, 7/G, Regione Amerique, 11020 Quart, Aosta Italy; 7 Azienda Sanitaria Locale Torino 3 di Collegno e Pinerolo, Via Martiri XXX Aprile, 30, 10093 Collegno, Torino, Italy

**Keywords:** ELISA, Europe, *Sarcoptes scabiei*, Sarcoptic mange, Serology, Seroprevalence, Switzerland, Wild boar

## Abstract

**Background:**

Sarcoptic mange has recently emerged in wild boar in Switzerland, raising the question of the origin of the infection. The main aim of this study was to assess the extent of exposure of the wild boar populations to *Sarcoptes scabiei* in Switzerland, prior to and after the detection of mange cases, to determine whether the mite has been recently introduced into the populations concerned. We performed a serological survey using a commercially available ELISA and 1056 archived blood samples of free-ranging wild boar from Switzerland. To facilitate the interpretation of the obtained data, we additionally estimated seroprevalence in wild boar populations of four other European countries (1060 samples), both from areas with confirmed clinical cases of mange and from areas without reported cases in wild boar. Lastly, we revised the evaluation of the commercial ELISA when used with wild boar sera.

**Results:**

Seropositive reactions were observed for samples from all five countries and from 15 of the 16 study areas. The obtained apparent seroprevalences ranged from 0.0% (0/82; 95% confidence interval [CI]: 0.0–4.4) to 17.4% (8/46; 95% CI: 7.8–31.4). Wild boar from study areas with known clinical cases and those ≤60 kg were four times more likely to be seropositive than wild boar from areas without reported cases and > 60 kg, respectively. Optical density values did not differ between the two types of study areas among seropositive samples but were significantly lower among seronegative samples from areas without than from areas with clinical cases. No difference was observed between the two sampling periods in Switzerland. The revised ELISA specificity was 96.8% (984/1017; 95% CI: 95.5–97.7) when wild boar from areas without history of mange were considered truly negative.

**Conclusions:**

Seropositivity to *S. scabiei* is more frequent and occurs over a larger geographic range than expected. Data suggest that the parasite is endemic within the wild boar populations of Switzerland and other European countries but that its presence is not necessarily associated with disease occurrence. Extrinsic factors which trigger disease emergence in infected populations remain to be investigated. The applied ELISA represents a promising tool for future studies.

**Electronic supplementary material:**

The online version of this article (10.1186/s12917-018-1430-3) contains supplementary material, which is available to authorized users.

## Background

Sarcoptic mange, caused by the burrowing mite *Sarcoptes scabiei*, is a highly contagious skin disease which occurs worldwide and affects a wide range of wild and domestic mammals as well as humans [1]. Mites are largely taxon-specific, however, transmissions across mammalian orders have been reported [2]. The disease is characterized by varying clinical signs and mortality, depending on a number of factors such as host species, season, immune status of the host, and presence of other diseases or nutritional imbalances [3, 4]. In wildlife, sarcoptic mange has repeatedly been investigated in various species developing severe lesions and undergoing high mortality [5–9]. By contrast, little is known on *S. scabiei* infections in wild boar (*Sus scrofa*), in which disease signs are relatively mild and mortality seems to be particularly uncommon [10].

In Switzerland, sarcoptic mange has been present in carnivores for several decades. It has progressively expanded to the whole Swiss territory in the red fox (*Vulpes vulpes*) population, and sporadic cases have also been observed in other carnivore species [11, 12]. By contrast, no cases had been recorded in wild ungulates until 2010, when mange was first diagnosed in wild boar in two Swiss regions distant from each other [10]. This disease emergence raised the question as to whether *S. scabiei* was recently introduced into the Swiss wild boar populations or was already present but had remained undetected. Since scanning surveillance has been shown to be not sensitive enough to detect mangy animals in naïve ungulate populations [6] and transmission of *S. scabiei* from carnivores to ungulates represents an unusual event [13], the possibility that *S. scabiei* was already endemic in wild boar in Switzerland deserved to be more carefully addressed.

Serology documents pathogen exposure independently of the presence of clinical signs or lesions, which makes it a useful tool to assess pathogen emergence when archived blood samples are available. Serology has been successfully used to study the dynamics of *S. scabiei* infections in free-ranging populations of wild carnivores and ruminants [14–18] but there are no such data on wild boar. A previous evaluation of an indirect commercial enzyme-linked immunosorbent assay (ELISA) developed for domestic pigs had revealed a test specificity of 80% when applied on wild boar samples from populations expected to be free of mange [19]. Nevertheless, it was uncertain whether this value was due to non-specific reactions, to cross-reactions with other mites, or to undetected mite infestations. Therefore, a comparison of seroprevalences obtained for populations considered as free of mange and those of areas with confirmed mange occurrence appeared necessary to better interpret the data.

The aims of this study were: (1) to assess the extent of exposure of the wild boar populations to *S. scabiei* in Switzerland prior to, and after the detection of mange cases, in order to determine whether this mite has been recently introduced into the populations; (2) to estimate the seroprevalence of *S. scabiei* in wild boar populations of several European countries, both from regions with confirmed clinical cases of mange and from regions without reported cases, to facilitate the interpretation of the data obtained for Switzerland. In addition, we proceeded to a re-estimation of the diagnostic specificity of the commercial ELISA when applied on sera from free-ranging wild boar, based on results obtained from areas without reported cases of mange.

## Material and methods

### Study areas, animals and samples

**Fig. 1:**
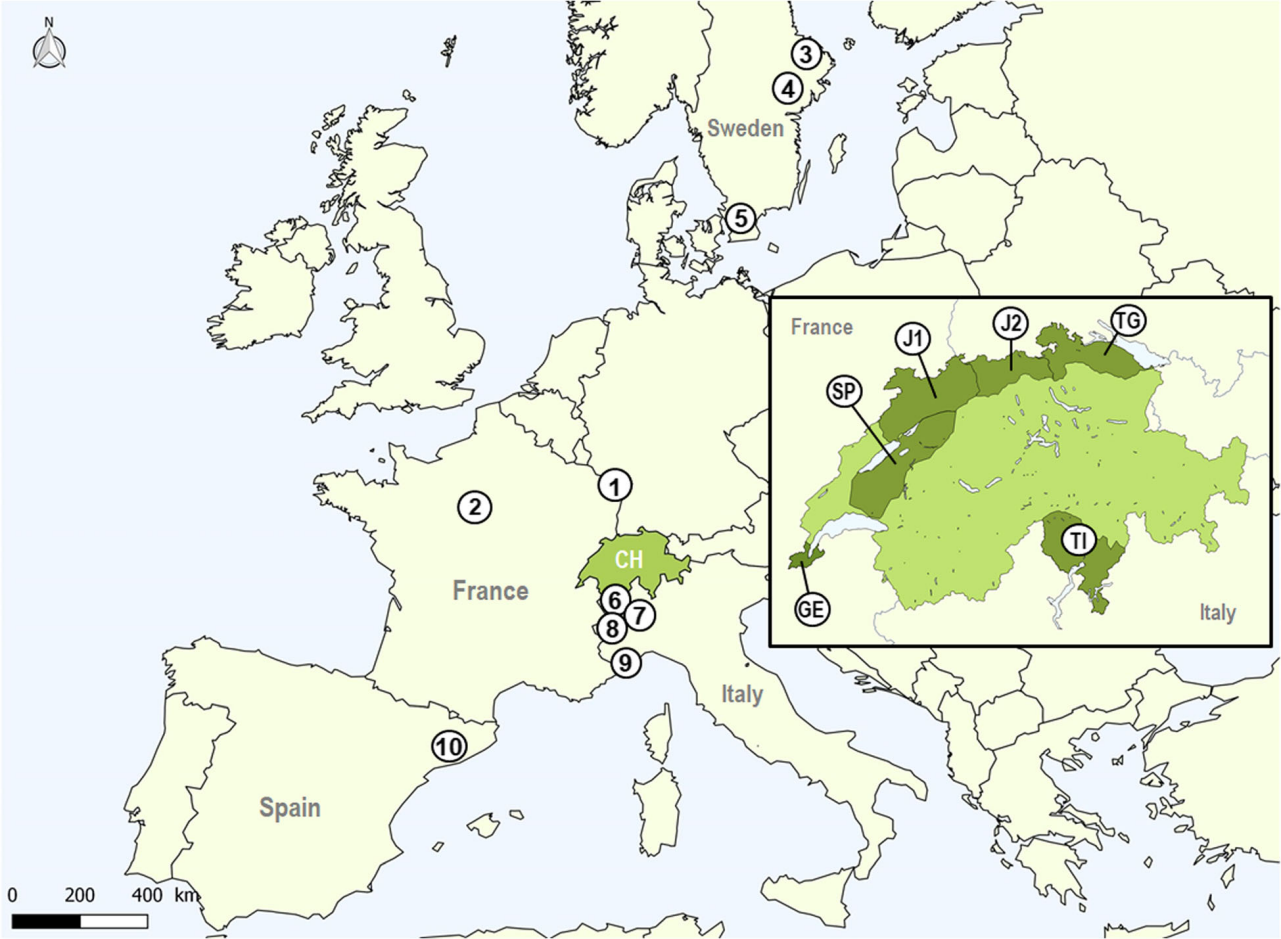
Map of western and northern Europe depicting the study areas. Numbers from 1 to 10 represent study areas in France, Sweden, Italy and Spain as indicated in Table 1. The framed map shows a close-up of Switzerland with the six Swiss study areas in dark green and labelled with the abbreviations indicated in Table 1

We used 2115 archived blood samples from wild boar from 16 study areas and five European countries, i.e., Switzerland, France, Italy, Sweden, and Spain (Additional file 1 and Fig. 1). The countries other than Switzerland were selected based on the availability of samples and the existence of a wild boar health monitoring program providing an insight into the mange status of the local population. Study areas were considered as epidemiological units because they were either located in distinct wild boar populations and/or distant from each other (> 60 km) and/or separated by major geographical barriers such as lakes and highways.

Target sample sizes were estimated per study area, using the free software WinEpiscope 2.0 (http://www.winepi.net/uk/index.htm), with an accepted error of 5% and level of confidence of 95%. Sample size for prevalence estimation in a population of unknown size was set to 139 for study areas with confirmed or suspected cases of sarcoptic mange (expected prevalence of 10%) and to 385 for areas supposed to be free of sarcoptic mange (expected prevalence of 50%).

In Switzerland, wild boar samples were available from 6 study areas (Table 1 and Fig. 1) and two independent populations, i.e., the southern population (Ticino) and the northern population (all other study areas). There were samples collected prior to (2008–2009; *n* = 239) and after (2010–2015; *n* = 816) the emergence of the first clinical cases of mange in wild boar. If the number of archived samples was larger than the target sample size, samples were arbitrarily selected to obtain, as far as possible, an even proportion between sexes and among age classes. In other countries, all samples were collected after 2010 and all available samples were used. The obtained sample size was below the target for most areas and the sample composition greatly differed among areas as concerns sex and age classes (Additional File 1), which reduced the precision of our prevalence estimates and limited the power of comparisons among areas. Since a major objective was to assess differences in prevalence between areas with and without reported cases of mange, areas were categorized according to their mange status, i.e., N-areas (no reported cases of mange; 6 areas, *n* = 1017), S-areas (suspected but unconfirmed cases of mange; 2 areas, *n* = 196), and C-areas (mange cases confirmed by mite identification; 8 areas, *n* = 902), and we used a modelisation approach to test the effect of the area’s mange status and the potential influence of individual factors such as sex and age on serological results (see Statistical Analyses).

**Table 1:** Study areas and results of the serosurvey. The total number of tested wild boar samples (*n*), total number of seropositive animals (pos), estimated seroprevalences in percent (Prev) and 95% confidence interval (CI) are given for each study area for all samples (total) and for wild boar ≤2 years old (young age class), together with the *p*-values of the binomial test comparing the estimated prevalence of each study area with Uppland (baseline). *P*-values < 0.05 are highlighted in bold. Note that although seroprevalence was found to be higher in young than adult wild boar in general, the reverse was observed in Skåne (46 youngs, 22 adults) and Södermanland (3 youngs, 26 adults). Wild boar are free-ranging in all areas but two: The National Domain of Chambord is fenced with high walls, in this area wild boar are regularly fed by game wardens, and until 2014 medicated food containing ivermectin was spread on feeding grounds; La Mandria is also delimited by physical boundaries (although occasional outbound and inbound dispersal movements of wild boar are not excluded)

Country	Study area	ID study area	Mange status^a^	Total samples		Young age class only			
				Prev in % (pos/*n*)	95% CI	*p*-value	Prev in % (pos/*n*)	95% CI	p-value
Switzerland	Geneva	GE	N	2.6 (10 / 389)	1.2–4.7	0.2955	3.3 (9 / 274)	1.5–6.1	0.6096
	Midlands	SP	N	0.1 (1/105)	0.0–5.2	1.0000	1.3 (1 / 77)	0.0–7.0	1.0000
	Jura 1	J1	C	12.7 (14 / 110)	7.1–20.4	**0.0021**	19.7 (14 / 71)	11.2–30.9	**0.0150**
	Jura 2	J2	N	3.4 (3 / 88)	0.7–9.6	0.2696	4.9 (3 / 61)	1.0–13.7	0.4964
	Thurgau	TG	S	1.3 (2 / 150)	0.2–4.7	0.7586	1.7 (2 / 115)	0.2–6.1	1.0000
	Ticino	TI	C	2.8 (6 / 213)	1.0–6.0	0.2823	4.0 (5 / 124)	1.3–9.2	0.5388
France	Vosges^b^	1	C	14.7 (28 / 191)	10.0–20.5	**0.0006**	15.0 (21 / 140)	9.5–22.0	**0.0378**
	Chambord^c^	2	S	17.4 (8 / 46)	7.8–31.4	**0.0004**	21.1 (4 / 19)	6.1–45.6	**0.0276**
Sweden	Uppland	3	N	0.0 (0 / 82)	0.0–4.4	–	0.0 (0 / 33)	0.0–10.6	–
	Södermanland	4	C	10.3 (3 / 29)	2.2–27.4	**0.0222**	0.0 (0 / 3)	0.0–70.8	NA
	Skåne	5	C	10.3 (7 / 68)	4.2–20.1	**0.0097**	8.7 (4 / 46)	2.4–20.8	0.2231
Italy	Aosta^d^	6	C	9.4 (6 / 64)	3.5–19.3	**0.0159**	23.8 (5 / 21)	8.2–47.2	**0.0139**
	Vercelli^e^	7	C	6.3 (1 / 16)	0.2–30.2	0.3598	8.3 (1 / 12)	0.2–38.5	0.5936
	La Mandria^f^	8	N	5.6 (10 / 179)	2.7–10.0	0.0665	5.7 (7 / 122)	2.3–11.5	0.3494
	Imperia^g^	9	C	6.2 (13 / 211)	3.3–10.3	**0.0473**	7.6 (10 / 132)	3.7–13.5	0.2211
Spain	Barcelona	10	N	1.2 (2 / 174)	0.1–4.1	0.8306	1.5 (2 / 137)	0.2–5.2	1.0000

^a^Three different mange status are considered: “non-reported” (N) in absence of known clinical cases, “confirmed” (C) if *Sarcoptes scabiei* was identified in skin samples from clinical cases, and “suspected” (S) if wild boar with suspicious skin lesions have occurred but the etiological role of *S. scabiei* was not confirmed (not investigated or not detected). In Thurgau, a few wild boar with focally extensive, well demarcated alopecia were documented by phototrapping but not submitted to veterinary examination. In the National Domain of Chambord, multiple wild boar with suspicious skin lesions were tested for mites in skin scraping material (light microscopy) or skin samples (histology, polymerase chain reaction) but *S. scabiei* was never found

^b^Parc Naturel Régional des Vosges du Nord (Vosges Department)

^c^National Domain of Chambord (Loir-et-Cher Department)

^d^Aosta Valley Region

^e^Vercelli Province (Piedmont Region)

^f^Parco Regionale La Mandria (Piedmont Region)

^g^Imperia (Liguria Region)

Samples were collected post mortem from wild boar hunted (*n* = 1955; all countries) or found dead (*n* = 49; Switzerland only), and from live animals (*n* = 111; Spain only). The age of the wild boar was determined according to body weight and coat color [20, 21] or based on the tooth wear of the inferior jaw [22–25]. We harmonized the dataset using three age classes according to the criteria of Hebeisen et al. (2008) [21]: 1) piglets (striped, < 6 months old, < 20 kg) and juveniles (reddish, 6–12 months old, between 20 and 40 kg), which were merged as one juvenile age class for our study; 2) subadults (black coat, 12–24 months old, > 40 kg and ≤ 60 kg); and 3) adults (black to silver coat, ≥ 2 years, > 60 kg).

Sampling was performed in the field by game-wardens, hunters, veterinarians or trained field technicians. In dead wild boar, blood or blood clots were taken from the heart, freshly open blood vessels, the retro-orbital sinus or body cavities. In live wild boar sampling was performed during capture by venipuncture directly from the heart prior to euthanasia. After collection, blood samples were transferred to the respective local laboratories, where the serum was separated by centrifugation, aliquoted and stored at − 20 °C until analysis.

### Laboratory analyses

We used the commercial indirect SARCOPTES-ELISA 2001® Pig (AFOSA GmbH, Dahlewitz bei Berlin, Germany), which uses *Sarcoptes* mites from pigs as antigen (whole mite antigen), according to the manufacturer’s instructions. We previously obtained a sensitivity of 75% and a specificity of 80% for this test when applied to wild boar sera, using pig control sera included in the kit [19]. Positive and negative predictive values of the ELISA obtained for wild boar sera were 0.56 and 0.94, respectively (C. Haas, unpublished data).

For the present study, we tested control sera from wild boar in addition to the pig sera provided in the kit. Negative wild boar control samples were collected on captive, healthy juvenile females from the Basel Zoo (Basel, Switzerland). Positive wild boar control samples were taken from a free-ranging, mangy juvenile female from the canton of Solothurn (Switzerland) with confirmed mite infestation [10]. Optical density (OD) ratios were calculated using the pig control sera according to the manufacturer’s instructions. Samples with doubtful results were re-tested up to six times.

Assuming that the study areas without reported mange cases are truly mange-free, we re-estimated the specificity of the ELISA as previously described [19], i.e., we calculated the proportion of samples with a negative result within the total number of tested samples expected to be truly non-infected (samples from N-areas; *n =* 1017). The 95% confidence interval was calculated according to the method of Wilson [26].

To compare the OD values obtained with different plates, a normalized OD value was calculated for each tested sample by subtracting the blank of the corresponding ELISA-plate from the crude OD value. For samples that were tested multiple times, the OD value of each run was normalized as described above and a single, final normalized OD value was obtained for each sample by calculating the mean of the multiple normalized OD values.

### Statistical analyses

Thirty-nine samples which remained doubtful despite retesting and were distributed among all animal categories (sex, age, area’s mange status) in seven study areas were considered as negative in the data analysis. All statistical analyses were performed using the R software version 3.3.2 (the R Project for statistical computing, available at https://www.R-project.org/; R Core Team 2016, R: A language and environment for statistical computing, R Foundation for Statistical Computing, Vienna, Austria).

Confidence intervals of apparent seroprevalences were estimated using the binom.test function and were therefore automatically computed according to Clopper and Pearson. We used generalized linear mixed models (glmm) with a logit link to test the effects of age (juvenile, subadult, adult), sex (male, female) and the area’s mange status (N, S, C) on the probability for a wild boar to be seropositive (dependent variable). We considered the serological status of wild boar as a bimodal variable (0/1): seronegative or doubtful results were encoded 0 and seropositive results were encoded 1. The effect of the study area (*n* = 16) was considered as a cluster random effect. Glmm models were computed using the function glmer from the package lme4 [27] and the dredge function of the package MuMIn (MuMIn, B. K. 2016, multi-model inference, R package version 1.15. 6. 2016.). We started with a “complete model” comprising the three explanatory variables and tested all simpler models. Model selection was based on the Akaike's Information Criterion (AIC) and the number of parameters: among the best models, i.e., with the lowest AIC and delta-AIC less than 2 (models fitting best the observed data), we retained the most parsimonious model (with less parameters) [28]. Lastly, parameters of the best model were estimated using the restricted maximum likelihood (REML) method [29] and their significance were tested using Wald tests [28].

The Fisher’s exact test (FET) was applied to compare seroprevalence between age classes (young and adult) in areas with and without confirmed mange cases (C and N status). The binomial test for comparison of two proportions (prop.test function) was used to compare the estimated seroprevalences of the different study areas and of the two sampling periods (before and after the first confirmed mange case, in three Swiss study areas for which older samples were available) with a baseline (a study area without reported clinical cases of mange and without seropositive reactions among the tested samples). The Kruskal-Wallis test and/or Wilcoxon test (with Bonferroni correction as appropriate) were used to test for significant differences among the normalized OD values of the seropositive and of the seronegative samples from the different study areas. We expected the average OD values of both positive and negative samples to be significantly lower in N-areas (nonspecific reactions) than in C-areas (*S. scabiei*-related reactions). The level of significance was set at *p* < 0.05.

## Results

The results of the serological survey are presented in Table 1. Seropositive reactions were observed for samples from all five countries and from all study areas except for Uppland in Sweden, and the highest apparent seroprevalence was recorded in the National Domain of Chambord (NDC; 14.4%, 95% CI: 7.4–31.0; Table 1). Regarding the considered predictor variables for seropositivity to *S. scabiei*, the best model retained the effect of wild boar age and area’s mange status but not the effect of sex (Table 2). Corresponding parameters are detailed in Table 3.

**Table 2:** Model comparison. List of the generated models and their characteristics. Each table line corresponds to one model. Df = degree of freedom, logLik = Log-likelihood. Model selection was done according to the Akaike’s Information Criterion (AIC). Models are ordered according to the AIC, with the best model on the top

Factor^a^			df	logLik	AIC	Delta AIC	AIC weight
Sex	Age	Mange status					
–	+	+	6	− 355.205718	722.411435	0	0.63956508
+	+	+	7	−354.924564	723.849129	1.43769374	0.31166893
–	+	–	4	− 360.163851	728.327702	5.91626678	0.03320349
+	+	–	5	−359.926631	729.853263	7.44182739	0.01548504
–	–	+	4	− 366.711672	741.423344	19.0119087	4.76E-05
+	–	+	5	−366.397265	742.79453	20.3830952	2.40E-05
–	–	–	2	− 371.194558	746.389117	23.9776816	3.97E-06
+	–	–	3	− 370.926502	747.853004	25.4415691	1.91E-06

^a^Factor involvement: + stands for inclusion and – for exclusion

**Table 3:** Parameters of the best model. Model parameters include the estimated logit coefficient (Estimate), the standard error of the coefficient (Std error), the z score (z-value) and the *p*-value of the Wald test (Pr(>|z|))

Variable	Estimate	Std error	z-value	Pr(>|z|)
Age (adult versus juvenile)	−1.42905	0.36330	−3.934	8.37e-05 ***
Age (subadult versus juvenile)	0.02359	0.23062	0.102	0.918534
Mange status (N vs. C)	−1.46035	0.41219	−3.543	0.000396 ***

**Fig. 2:**
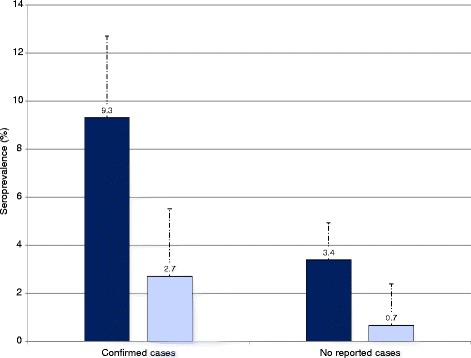
Histogram of the apparent seroprevalences (in %) according to age classes. The young class (juveniles and subadults, ≤ 60 kg) is represented in deep blue and the adult class in light blue. The vertical dotted lines over the bars represent the upper limit of the corresponding 95% confidence intervals. Seroprevalence data are given for areas pooled according to their mange status (“confirmed cases”, i.e., *Sarcoptes scabiei* was identified in skin samples; and “no reported cases”). The two areas with suspected but unconfirmed cases were excluded from the graph (National Domain of Chambord in France: likely cases, high apparent seroprevalence, 8 seropositive samples; Thurgau in Switzerland: doubtful cases, low apparent seroprevalence, 2 seropositive samples)

Juveniles were significantly more often seropositive than adults while no significant difference was found between juveniles and subadults (Table 3), and these two age classes were pooled as a “young” age class (≤ 60 kg). Young wild boar were four times more likely to be seropositive than adults (OR young/adult = 4.2, 95% CI: 2.1–8.3; estimate = 1.4406, standard error = 0.3452, z-value = 4.173, Pr(>|z|) = 3.01e-05 ***). This difference between the young and adult age classes was observed both in C- and N-areas (FET, *p* < 0.001 and *p* < 0.01, respectively; Fig. 2). Wild boar from N-areas (no clinical cases reported) showed a significantly lower seroprevalence (2.6%, 26/1017, 95% CI: 1.7–3.7) than those from C-areas (confirmed clinical cases; 8.6%, 78/902, 95% CI: 6.9–10.7; OR 4.3, 95% CI: 1.9–9.7).

Tentative comparison among study areas revealed that all areas with apparent seroprevalence < 1.3% were N-areas, while areas with seroprevalences ≥ 6.2% were C-areas except for the NDC (S-area). All areas ≥ 6.2% showed seroprevalences significantly higher than Uppland (N-area, baseline) except Vercelli, for which we had only a low sample size (6.3%, 95% CI: 3.3–10.3, *n* = 16). Seroprevalences from 1.3% to < 6.2% did not significantly differ from that in Uppland and corresponded to a mixture of areas with different mange status: Thurgau (S-area), Ticino (C-area), Geneva, Jura 2 and La Mandria (N-areas; Table 1).

Regarding the sampling periods, apparent seroprevalences for the Swiss areas Geneva (N-area) and Ticino (C-area) did not reveal differences from the seroprevalence in Uppland in any of the two periods, while the seroprevalence of Jura 1 (C-area) did significantly differ from that in Uppland in both periods (Table 4), i.e., we found no indication that the emergence of clinical cases in Jura 1 and Ticino was associated with a change in local seroprevalence.

**Table 4:** Estimated seroprevalences in the two sampling periods in three study areas in Switzerland. Seroprevalences (Prev) are given in percent (%) with the number of positive samples (pos) and total number of samples (*n*) in parenthesis, the 95% confidence interval (CI) and the *p*-value of the binomial test applied to compare local seroprevalences with that of Uppland (baseline, see Table 1). This information is provided both for all samples and for the young age class (≤ 60 kg) only. *P*-values < 0.05 are highlighted in bold

Study area	Mange status^a^	Period	Total samples			Young age class only		
			Prev in % (pos/*n*)	95% CI	*p*-value	Prev in %, (pos/*n*)	95% CI	*p*-value
Geneva	N	1	3.6 (6/167)	1.3–7.7	0.1944	4.6 (5/108)	1.5–10.5	0.4710
	N	2	1.8 (4/222)	0.5–4.6	0.5115	2.4 (4/166)	0.7–6.1	0.8245
Jura 1	N	1	18.2 (2/11)	2.3–51.8	**0.0052**	25.0 (2/8)	3.2–65.1	**0.0423**
	C	2	12.1 (12/99)	6.4–20.2	**0.0031**	19.0 (12/63)	10.2–30.9	**0.0185**
Ticino	N	1	5.3 (3/57)	1.1–14.6	0.1318	8.3 (2/24)	1.0–27.0	0.3375
	C	2	1.9 (3/156)	0.4–5.5	0.5141	3.0 (3/100)	0.6–8.5	0.7411

^a^N no reported clinical cases, C confirmed clinical cases (*S. scabiei* was identified in skin samples)

Regarding the OD values of seropositive samples (*n* = 114), there was no significant difference between C-areas and N-areas. Among seronegative wild boar (including doubtful results; *n* = 1192), OD values in C-areas were significantly higher than values in N-areas (*p* < 0.05) and the OD values of wild boar from the NDC (*n* = 38) differed from N-areas (*p* < 0.005) but not from C-areas. Values from Thurgau (*n* = 148) did not differ from any group. Furthermore, values of seronegative young wild boar were significantly higher than those of seronegative adults (*p* < 0.001).

Repeated testing of the positive and negative wild boar control sera always yielded positive and negative results, respectively. The re-estimated specificity of the test was 96.8% (95% CI: 95.5–97.7) when animals from all presumably *Sarcoptes*-free areas were pooled (984 negative samples out of 1017 tested samples from N-areas; doubtful results excluded); when N-areas were individually considered, the proportion of negative samples reached 100% for Uppland in Sweden (*n* = 82).

## Discussion

Seropositive wild boar were detected both in areas with confirmed mite presence (C-areas) and in areas where wild boar were presumed to have not been exposed to *S. scabiei* (N-areas). However, there was a relationship between the detection of clinical cases and the level of seroprevalence. Apparent seroprevalences ≥ 6.2% were all found in C-areas, with the exception of the NDC in France (suspected unconfirmed cases, S-area), which displayed the highest prevalence of all study areas. Another similarity between C-areas and the NDC was that seronegative wild boar had a significantly higher mean OD value than those from N-areas, which may be due to rising titers in recently exposed wild boar. These observations together with the repeated observation of mange-like clinical signs in piglets in the NDC is consistent with an endemic presence of *S. scabiei* in this study area despite the unsuccessful mite detection. As wild boar from this population were occasionally administered medicated food with ivermectin via feeding grounds (Table 1), a previous treatment may have eliminated the parasite [30] before the full resolution of the skin lesions. Alternatively, the clinical examination of the piglets at capture may have occurred during the spontaneous healing phase of the disease, after successful elimination of the mite [1, 3, 4], or mite detection failed due to the poor sensitivity of skin scraping and PCR in cases with mild lesions, in which mites are few [31–34].

By contrast, estimated seroprevalences < 6.2% were associated with an absence of reports of mange cases (N-areas), except for the Swiss areas Ticino (C-area) and Thurgau (S-area). In Ticino, mange lesions were limited to the ears and detected in only two wild boar [10]. Together with our serological results, this indicates that *S. scabiei* can be present at low prevalence in wild boar populations. In line with this, mean OD values of seropositive samples did not differ between N- and C-areas. Furthermore, these data suggest that areas with similar or even higher seroprevalences than Ticino such as Geneva, Jura 2 and La Mandria may also be infected despite the lack of case reports. As for Thurgau, the particularly low prevalence recorded (1.3%, 95% CI 0.2–4.7) suggests that the observed skin lesions [10] may have had a different etiology than *S. scabiei*, such as *Demodex* sp. [1, 32, 35]. However, this cannot be definitely elucidated solely by serology.

### Age-related differences

We did not find an effect of the sex on seropositivity, in accordance with previous observations in other wildlife species [6, 7, 36, 37] but young wild boar were more likely to be seropositive than adults. This seropositivity pattern among age classes was the same in C- and N-areas (Fig. 2), which does not support the hypothesis that seropositive wild boar in areas without reported disease cases were due to unspecific reactions.

Higher seroprevalence in young wild boar suggests a first exposure to mites early in life. Higher OD values in young than adult seronegative animals may be due to rising titers in recently exposed wild boar and decreasing titers in animals having overcome the infection. Antibodies to *S. scabiei* persist up to one year after elimination of the mites [38, 39], i.e., antibodies may not be detected in adult wild boar unless re-infection has occurred. In domestic pigs, sheep and rabbits, a second infection with *S. scabiei* can be characterized by lower IgG antibodies and higher IgE antibodies levels than during the first infection [1, 32, 33, 40, 41]. Thus, adult wild boar may be less often seropositive than juveniles and subadults because the ELISA detects IgG antibodies (instead of IgE) and adult wild boar are more likely to have been repeatedly exposed to mites than young animals. These results suggest that the parasite is endemic in the sampled populations and are consistent with the perception by field operators that wild boar are mostly able to cope with *S. scabiei* infections and not prone to develop fatal disease [10].

### Parasite versus disease occurrence

Our data indicate that *S. scabiei* may be distributed over a larger area than inferred when considering only wild boar with clinical signs of mange. Similarly, antibodies were detected in healthy chamois (*Rupicapra r. rupicapra* and *R. pyrenaica parva*) from apparently mange-free areas [6, 16]. Healthy carriers of *S. scabiei* have been reported at least in pigs, Spanish ibex (*Capra pyrenaica*) and humans [1, 32, 42–44]. Furthermore, it has been documented that after a peak of sarcoptic mange in a wild ungulate population, when most sensitive animals have disappeared, the mite continues to spread among survivors despite the lack of visible clinical signs [6, 7, 15], and the derived recovering population is remarkably more resistant to disease [6]. Under such newly established endemic conditions, observed seroprevalences are moderate [16]. Similarly, there has been an increase of seropositive but apparently healthy red foxes derived from a population affected by sarcoptic mange for a long time [45]. Thus, the detection of wild boar seropositive to *S. scabiei* in a population without known clinical cases of mange may not only be due to false-positive reactions but could also reveal the presence of the parasite in the population and the existence of a host-parasite equilibrium. Since the severity of clinical signs is related to the ability of the host to control mite infestation [4, 36, 46, 47], animals without lesions are expected to be less infectious than animals with a clinical expression, which may explain the limited distribution of the parasite in the population, i.e., the lower seroprevalence.

We have shown that ELISA reactors were similarly prevalent in different cohorts collected in Switzerland before and after disease emergence, which is also consistent with an endemic situation. Sample size in period 1 was limited and results need to be taken with caution. Nevertheless, the apparent emergence of clinical cases may have been due to a better detection thanks to improved monitoring techniques (phototrapping) and/or increased disease awareness among hunters and wildlife professionals in recent years, or due to a true disease emergence triggered by environmental factors. Host population density, meteorological conditions and/or food availability are factors believed to influence the course of infections with *S. scabiei* [6, 9, 32].

### ELISA performance

The specificity of a diagnostic test in wildlife is particularly difficult to determine as it requires truly non-infected animals. Unless the individuals were born in highly controlled captive settings and their health status closely monitored since birth, exposure to pathogens cannot be completely ruled out. We previously estimated the specificity of the SARCOPTES-ELISA 2001® Pig at 80% when applied to wild boar sera, and we interpreted positive results obtained from free-ranging wild boar from a supposedly mange-free population (Geneva) as false positive [19]. Here we obtained a specificity of 96.8% (95% CI 95.5–97.7) when seronegative samples from all N-areas were considered true negative and we raise doubts as concerns the mite-free status of Geneva and some other N-areas. This value of 96.8% is considerably higher than our first estimation and closer to those generally reported for commercially available or “in-house” ELISAs [31, 36, 48]. In view of the results from the present serosurvey, we propose that the specificity is higher than previously estimated when applied to wild boar sera. Nevertheless, it would be useful to develop complementary laboratory tests such as a Western Blot to confirm the presence of specific *S. scabiei* antigens and obtain more accurate serological results in the future. An estimation of the sensitivity and specificity of the ELISA may also be performed with a Bayesian approach.

## Conclusions

This study suggests that *S. scabiei* is more widely distributed in free-ranging wild boar populations than previously assumed and that the parasite has been endemic in Switzerland already before the first disease cases were observed. The awareness of hunters and wildlife professionals needs to be further promoted to obtain a better picture of the distribution of clinical cases and to monitor potential changes in disease occurrence. The applied ELISA represents a promising tool for future surveys.

## Additional file

Additional file 1: Sample composition and sampling periods for each study area. Sample size is given as total per study area and according to sex and age (J = juvenile, S = subadult, A = adult and Un = unknown) of the sampled wild boar. (DOCX 21 kb)

## **Abbreviations**

AIC: Akaike’s Information Criterion
CI: confidence interval
df: degree of freedom
ELISA: Enzyme-linked immunosorbent assay
FET: Fisher’s exact test
Fig: Figure
FIWI: Centre for Fish and Wildlife Health
glmm: generalized linear mixed model
NDC: National Domain of Chambord
OD: optical density
OR: odds ratio
REML: restricted maximum likelihood
*S. scabiei*: *Sarcoptes scabiei*
Std: standard
