# Supplementary material for: Serological survey in wild boar (Sus scrofa) in Switzerland and other European countries: Sarcoptes scabiei may be more widely distributed than previously thought
Source: BMC Vet Res. 2018 Mar 27;14:117. doi: 10.1186/s12917-018-1430-3 (PMC5872548; doi:10.1186/s12917-018-1430-3)
Supplement: Supplementary file 1 — Sample composition and sampling periods for each study area. Sample size is given as total per study area and according to sex and age (J = juvenile, S = subadult, A = adult and Un = unknown) of the sampled wild boar. (DOCX 21 kb) [file 12917_2018_1430_MOESM1_ESM.docx]

**Additional file 1. Sample composition and sampling periods for each study area.** Sample size is given as total per study area and according to sex and age (J= juvenile, S=subadult, A=adult and U=unknown) of the sampled wild boar.

| **Country** | **Study area** | **Sampling**  **period**  **(month/year)** | **Wild boar** | | | | | | | | | | | |  |
| --- | --- | --- | --- | --- | --- | --- | --- | --- | --- | --- | --- | --- | --- | --- | --- |
|  |  |  | **Females** | | | | **Males** | | | | **Unknown sex** | | | | **Total** |
|  |  |  | **J** | **S** | **A** | **U** | **J** | **S** | **A** | **U** | **J** | **S** | **A** | **U** |  |
| Switzerland | Geneva | 09/09 – 03/15 | 65 | 67 | 45 | 11 | 60 | 82 | 50 | 9 | 0 | 0 | 0 | 0 | 389 |
|  | Midlands | 01/12 – 05/15 | 16 | 15 | 11 | 0 | 28 | 18 | 17 | 0 | 0 | 0 | 0 | 0 | 105 |
|  | Jura 1 | 12/08 – 12/14 | 18 | 17 | 18 | 0 | 23 | 13 | 21 | 0 | 0 | 0 | 0 | 0 | 110 |
|  | Jura 2 | 02/12 – 01/15 | 21 | 12 | 17 | 0 | 23 | 5 | 10 | 0 | 0 | 0 | 0 | 0 | 88 |
|  | Thurgau | 07/12 – 02/15 | 25 | 31 | 9 | 0 | 32 | 27 | 26 | 0 | 0 | 0 | 0 | 0 | 150 |
|  | Ticino | 01/09 – 01/15 | 33 | 32 | 39 | 1 | 26 | 33 | 45 | 4 | 0 | 0 | 0 | 0 | 213 |
| France | Chambord | 01/15 | 5 | 5 | 6 | 5 | 8 | 1 | 6 | 10 | 0 | 0 | 0 | 0 | 46 |
|  | Vosges | 02-05/15 | 36 | 16 | 0 | 14 | 64 | 23 | 0 | 33 | 1 | 0 | 0 | 4 | 191 |
| Sweden | Uppland | 01/13 – 12/15 | 15 | 18 | 40 | 7 | 0 | 0 | 0 | 2 | 0 | 0 | 0 | 0 | 82 |
|  | Södermanland | 01/13 – 05/14 | 1 | 2 | 8 | 16 | 0 | 0 | 0 | 2 | 0 | 0 | 0 | 0 | 29 |
|  | Skåne | 11-12/13, 11-12/14,12/15 | 29 | 17 | 19 | 3 | 0 | 0 | 0 | 0 | 0 | 0 | 0 | 0 | 68 |
| Italy | Aosta | 2011 – 2016 | 4 | 6 | 26 | 0 | 6 | 4 | 18 | 0 | 0 | 0 | 0 | 0 | 64 |
|  | Vercelli | 2011 – 2016 | 3 | 2 | 3 | 0 | 3 | 4 | 1 | 0 | 0 | 0 | 0 | 0 | 16 |
|  | La Mandria | 2011 – 2012 | 13 | 42 | 25 | 1 | 12 | 54 | 18 | 0 | 1 | 0 | 2 | 11 | 179 |
|  | Imperia | 09-11/ 14 | 21 | 39 | 34 | 0 | 37 | 35 | 45 | 0 | 0 | 0 | 0 | 0 | 211 |
| Spain | Barcelona | 06/12 – 04/16 | 38 | 22 | 21 | 0 | 40 | 33 | 16 | 0 | 0 | 0 | 0 | 4 | 174 |
| Total | -- | -- | 343 | 343 | 321 | 58 | 362 | 332 | 273 | 60 | 2 | 0 | 2 | 19 | 2115 |

^1^HS=Hunting season
